# Dual Trajectories of Serum Brain-Derived Neurotrophic Factor and Cognitive Function in People Living with HIV

**DOI:** 10.21203/rs.3.rs-4307577/v1

**Published:** 2025-04-14

**Authors:** HENRY MICHAEL, Antony Rapulana, Theresa Smit, Njabulo Xulu, Sivapragashini Danaviah, Suvira Ramlall, Frasia Oosthuizen

**Affiliations:** University of KwaZulu-Natal; Africa Health Research Institute; Africa Health Research Institute; Africa Health Research Institute; Eduvos; University of KwaZulu-Natal; University of KwaZulu-Natal

**Keywords:** HIV/AIDS, Cognition, Brain-derived neurotrophic factor, Group-based trajectory modelling, sub-Saharan Africa

## Abstract

This study aimed to identify the interrelationships between mature BDNF (mBDNF), precursor BDNF (proBDNF) trajectories, and cognitive performance in individuals with HIV from sub-Saharan Africa over 96 weeks following antiretroviral therapy (ART) initiation. Using data from 154 participants in the ACTG 5199 study (ClinicalTrials.gov NCT00096824, 2005–06-23) in Johannesburg and Harare (2006–2009), we measured serum mBDNF and proBDNF levels via ELISA and assessed cognitive performance with neuropsychological tests. Group-based trajectory modelling indicated two mBDNF trajectories—“Stable Ascent” (83.9%) and “Peak with Gradual Decline” (16.1%)—and two proBDNF trajectories—“Gradual Increase” (85.7%) and “Gradual Decline” (14.3%). These were linked to three cognitive trajectories: “Low Baseline-Slow Improvement,” “Gradual Improvement,” and “Late Surge.” The “Stable Ascent” mBDNF group showed a significant probability of “Gradual Improvement” (68%) in cognitive performance and a “Late Surge” (9.5%). In contrast, the “Peak with Gradual Decline” mBDNF trajectory saw no “Late Surge.” A “Gradual Increase” in proBDNF corresponded to a 67.7% chance of “Gradual Improvement” in cognition. Findings suggest BDNF isoforms as potential biomarkers for cognitive interventions in HIV, emphasizing that stable or increasing BDNF levels post-ART are linked to favourable cognitive outcomes. Further research is needed to develop BDNF-based cognitive health strategies to improve outcomes for people with HIV.

## INTRODUCTION

Despite a 69% drop in HIV-related deaths since 2004 due to wider antiretroviral therapy (ART) access[1], individuals with HIV still face significant risks of comorbidities, notably HIV-associated neurocognitive impairment[2]. Affecting 43% of the global HIV community[3] and 45% in sub-Saharan Africa[4], this condition stems from various factors such as chronic immune activation, inflammation, ART side effects, opportunistic infections, social determinants, and stigma[5–7]. HIV-associated neurocognitive impairment compromises treatment adherence and quality of life and increases healthcare demands and costs[8]. This underscores the critical need for the discovery and targeting of new biological pathways, and the development of biomarkers for early detection, prevention, tailored interventions, and monitoring treatment effectiveness.

Brain-derived neurotrophic factor (BDNF) is increasingly recognized as a potential biomarker for cognitive changes in various neuropathologic conditions due to its critical roles in synaptic plasticity, neurogenesis, and neuronal differentiation[9]. Extensive evidence from the general population highlights the benefits of mature BDNF (mBDNF) on cognitive and motor learning via mechanisms like long-term potentiation[10]. Conversely, mBDNF’s precursor, proBDNF, is associated with long-term depression and apoptotic pathways, suggesting a nuanced role in brain function[10, 11]. Throughout this article, the term “BDNF” without a specific prefix is used to refer either to the overall category of BDNF-related proteins or in reference to instances where distinctions among the protein’s forms are not made clear in cited studies.

Although longitudinal research on BDNF and cognitive function in HIV is limited, various cross-sectional studies have examined the relationship between BDNF levels and cognition among people living with HIV, with inconsistent results. For example, an Italian study reported that higher BDNF levels in serum were linked to slower performance on the Grooved Pegboard Test[12]. Conversely, a U.S. study found that increased serum BDNF levels improved learning and delayed recall in older African-American individuals living with HIV[13]. Another study highlighted a nuanced relationship between plasma BDNF levels and cognitive performance in individuals with HIV, suggesting that moderate, not high, BDNF levels might be protective[14].

The variability in the impact of BDNF on cognitive function across individuals living with HIV may stem from previous studies’ lack of distinction between the effects of mBDNF and its precursor, proBDNF. This oversight complicates comparisons with the broader body of literature. Additionally, inadequate consideration of temporal dynamics, potential confounders, cognitive domains assessed, and variability in sample sites and types has likely contributed to inconsistent findings, both within HIV research and more generally[15].

While mixed-effect models are adept at analyzing longitudinal data, they presuppose a uniform trajectory across individuals, which may simplify the intricate dynamics between BDNF and cognitive performance [16]. Group-based trajectory modeling (GBTM) addresses this limitation by allowing for the identification of distinct subgroups within the population that share similar patterns over time. This method is particularly advantageous for capturing heterogeneity and uncovering co-occurring or divergent trajectories of biomarkers and cognitive outcomes, offering deeper insights into underlying processes. By leveraging GBTM, this study aims to unravel the intertwined trajectories of mBDNF, proBDNF, and cognition, providing a more nuanced understanding of their relationships.

Towards this end, our study adopts Group-based Dual Trajectory Modeling (GBDTM) to explore the intertwined trajectories of mBDNF, proBDNF, and cognitive performance in adults living with HIV in sub-Saharan Africa. GBDTM enhances traditional univariate group-based trajectory modelling by simultaneously examining two distinct outcomes and their evolution over time, thereby capturing their potential comorbidity or heterotypic continuity[17]. This method, rooted in finite growth mixture modelling, identifies groups of individuals sharing similar patterns over time, offering a nuanced understanding of the interplay between biological markers and cognitive performance[18]. Although previous research has shed light on BDNF’s impact on patient outcomes[19, 20] and complex cognitive phenotypes in HIV[21–24], it has not linked these elements. Our study is pioneering in applying GBDTM to unravel the concurrent development trajectories of mBDNF, proBDNF, and cognition among individuals with HIV, marking a significant step forward in understanding their dynamic relationship.

In this study, we posed four research questions: (1) What are the distinct trajectories of mBDNF, proBDNF, and cognitive performance in a cohort of people living with HIV over 96 weeks post-ART initiation? (2) What proportion of the cohort follows each identified trajectory? (3) What sociodemographic and clinical factors influence the trajectories of mBDNF and proBDNF? (4) How are the trajectories of mBDNF and proBDNF linked to changes in cognitive performance when analyzed together? We hypothesize that an upward trajectory in cognitive performance will correlate with rising levels of mBDNF and a decrease in proBDNF levels.

## METHODS

This study was a secondary analysis of serum specimens and data from the Johannesburg and Harare sites of the ACTG A5199, the International Neurological Study (INS) from 2006–2009. The ACTG 5199 study evaluated the neurological and neuropsychological effects of three randomly assigned antiretroviral regimens in resource-limited settings and the study methods have been detailed in previous reports[25–27].

### Cohort description

This analysis focused on ART-naïve individuals with CD4 counts under 300, who were part of the ACTG A5175 multinational trial assessing the efficacy and safety of antiretroviral treatments (ClinicalTrials.gov, NCT00084136) [28]. Participants were required to have a Karnofsky performance score of at least 70 and no more than a week’s experience with antiretroviral therapy prior to the study’s commencement. Those with recent substance or alcohol abuse, severe psychiatric illnesses, hospitalization within the fortnight preceding study entry, or any condition likely to affect study participation or results interpretation were excluded. Follow-up included neurocognitive and neurological evaluations every 24 weeks over a period of up to 192 weeks, with this study reporting on data and specimens collected within the first 96 weeks.

### Measurements

#### Quantitative ELISA Analysis of Serum mBDNF and proBDNF Levels

Quantitative analysis of serum mBDNF and proBDNF levels was conducted using a specific enzyme-linked immunoassay (ELISA) kit, adhering to the standardized protocols provided by Biosensis Pty Ltd., Thebarton, SA, Australia. To maintain consistency and reduce variability between assays, samples, which were preserved at −70°C, were tested in pairs on the same ELISA plate. The intra-assay variability for mBDNF and proBDNF was notably low, recorded at 2.7% and 2.2% respectively, confirming the precision of the measurements. Additionally, the laboratory technicians analyzing the samples were kept unaware of the participants’ clinical and cognitive status to eliminate any potential for bias in the results.

#### Neuropsychological tests

Data collected as part of the ACTG study was accessed and used in this analysis. During the ACTG study, participants underwent a battery of four neuropsychological tests – grooved pegboard (both hands)[29], finger tapping (both hands)[30], timed gait[31] and semantic verbal fluency tests[32]. These tests were administered primarily by physicians, with training and quality assurance conducted annually, complemented by biannual centralized training sessions. Sites underwent initial quality evaluations before full enrollment, ensuring readiness. Data collection was standardized through translated and back-translated case forms to resolve inconsistencies. Ongoing monitoring included automated range checks and follow-ups on outliers, safeguarding data accuracy and reliability[26, 27]. The neuropsychological assessments were conducted at baseline and then followed up every 24 weeks. We utilized neuropsychological data at baseline, 24, 48 and 96 weeks. Test scores were normalized into z-scores, utilizing demographic adjustments (site, age, sex, and education-adjusted) based on data from the International Neurocognitive Normative Study ACTG A5271, encompassing a wide array of at-risk populations[33]. These z-scores were then averaged to formulate a composite cognitive z-score, which serves as a measure reflective of cognitive performance, with higher scores denoting higher cognitive performance.

#### Predictors

To investigate predictors of trajectory group membership, we analyzed key demographic and clinical variables. These included age (in years), sex (male/female), years of education, study site (Harare/Johannesburg), baseline CD4 count (cells/mm^3^), baseline CD8 count (cells/mm^3^), CD4/CD8 ratio, and log-transformed plasma HIV RNA levels. These variables were chosen based on their relevance to HIV-related neurocognitive outcomes and their potential to influence neurotrophic factors. Following the identification of trajectory groups, regression models were employed to determine the associations between these predictors and trajectory group membership.

### Statistical analysis

We included participants who had completed at least two neuropsychological assessments across the study period. To explore distinct patterns over time, we applied group-based trajectory modelling (GBTM) to each variable (log-transformed serum mBDNF, log-transformed proBDNF, and the composite cognitive z-score) using an iterative approach to determine the optimal number and shape (linear, quadratic, or cubic) of trajectories[18, 34]. Log transformation was applied to mBDNF and proBDNF values to normalize their distributions prior to modelling. GBTM assumes that the population comprises discrete subgroups that follow distinct developmental trajectories. The selection of the best-fitting model for mBDNF, proBDNF, and the composite cognitive z-sore was grounded in a comprehensive evaluation of model fit diagnostics and a set of criteria aimed at minimizing the risk of identifying spurious groups [35]. These criteria included the Bayesian Information Criterion (BIC) for model fit, with higher BIC values signifying a better fit; an Average Posterior Probability (APP) of group assignments above 0.70; a minimum group membership probability of about 5% of the sample; odds of correct classification exceeding 5; tight 99% confidence intervals around estimated group memberships; and evidence of statistically significant groups (P < 0.05), along with the requisites of graphical clarity, clinical relevance, and theoretical interpretability.

Subsequently, participants were categorized into trajectory groups based on their maximum posterior probability derived from the final models. We conducted descriptive analyses to outline baseline characteristics of the cohort and further analyzed differences in demographic and HIV-related variables across mBDNF and proBDNF trajectory groups using Wilcoxon rank-sum tests for continuous variables and chi-square tests for categorical variables. Following these univariate comparisons, multivariable logistic or multinomial regression was utilized to ascertain significant predictors of group membership, setting a significance threshold at P < .05.

Additionally, dual trajectory models elucidated the relationship between BDNF levels and cognitive performance, presenting conditional and joint probabilities to highlight the mutual influence of mBDNF and the composite cognitive z-score, as well as proBDNF and the composite cognitive z-score trajectories[16]. This comprehensive analysis leverages the “lcmm” package[36] within R software version 4.3.1, facilitating an in-depth exploration of the longitudinal relationships between neurotrophic factors and cognitive trajectories in our cohort. The annotated R code for the dual trajectory analysis is available in Supplementary Material S1.

## RESULTS

Of the 158 participants with baseline neuropsychological data and serum samples, 4 were excluded due to lack of follow-up, yielding a baseline analytical sample of 154 participants (Supplementary Material S2). In our statistical analysis, we incorporated data from participants who completed at least two neuropsychological assessments. Missing data were assessed for key variables including serum mBDNF, proBDNF, and the composite cognitive z-score. Specifically, missing values for mBDNF ranged from 1 at baseline to 27 at week 96, while for proBDNF, missing values ranged from 11 at week 24 to 47 at week 96. Notably, no missing values were observed for the composite cognitive z-score. The models incorporated all available data using maximum likelihood estimation, a robust approach for longitudinal analyses, ensuring reliable and unbiased parameter estimation despite the presence of missing data[37].

The median baseline age was 35 years with an interquartile range (IQR) of 10 years; the majority, 66.2%, were female, and the median educational attainment was 11 years (IQR: 3). The baseline median cognitive z-score was 0.19 with an IQR of 1.01 [Table1, Panel A].

### Trajectories of Serum mBDNF

**Figure 1:**
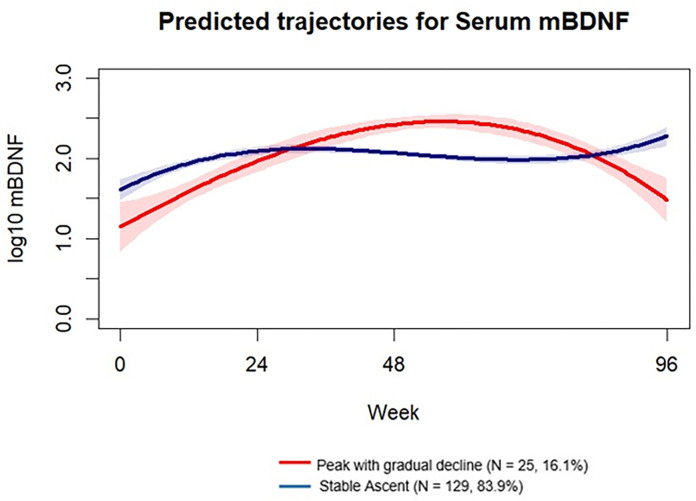
Predicted trajectory groups for serum mBDNF. “Peak with gradual decline” group (16.1 %) is represented in red; “Stable Ascent” group (83.9%) is represented in blue. The shaded regions around the trajectories represent the 95% confidence intervals, illustrating the uncertainty or variability in the model’s predictions over time. Narrower shading indicates greater confidence in the predicted values. Abbreviation: mBDNF, mature brain-derived neurotrophic factor

Based on fit indices and our a priori criteria, we identified two trajectories of mBDNF over 96 weeks post-ART. The best-fitting model was a two-group cubic model, with trajectory shapes that captured a “Stable ascent” (N = 129, 83.9%) and a “Peak with gradual decline” (N = 25, 16.1%) (Figure 1). Supplementary material S3 provides detailed information on model fit indices and diagnostics of assignment accuracy. Table 1 (Panel B) presents baseline descriptive statistics for the mBDNF trajectory groups. Participants in the stable ascent group tended to be younger, with lower CD8 cell counts at baseline and higher mBDNF levels than those in the “peak with gradual decline” group. However, there were no significant differences between the groups regarding years of education, sex, plasma viral load, CD4 count, and baseline cognitive performance.

Furthermore, the results of multivariable logistic regression analysis, as shown in Supplementary material S4, indicate that baseline age, CD8 count, and baseline serum mBDNF were significant predictors of mBDNF trajectory group membership.

### Trajectories of Serum mBDNF

**Figure 2:**
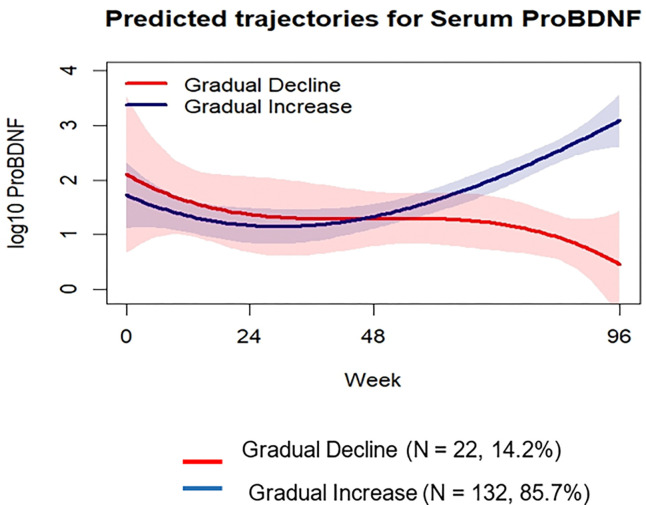
Predicted trajectory groups for serum proBDNF. “Gradual Decline” group (14.2 %) is represented in red; “Gradual Increase” group (85.7%) is represented in blue. The shaded regions around the trajectories represent the 95% confidence intervals, illustrating the uncertainty or variability in the model’s predictions over time. Narrower shading indicates greater confidence in the predicted values. Abbreviation: proBDNF, precursor brain derived neurotrophic factor

We identified two trajectories of serum proBDNF over 96 weeks post-ART that best fit the data. The optimal model was a two-group cubic model, resulting in trajectories labelled as “Gradual Increase” (N = 132, 85.7%) and “Gradual Decline” (N = 22, 14.3%) (Figure 2). Supplementary material S5 provides detailed information on model fit indices and diagnostics of assignment accuracy. Table 1 (Panel C) presents baseline descriptive statistics for the proBDNF trajectory groups. Participants were only significantly different (p < 0.05) based on study site. Furthermore, study site remained significant (Log odds −0.07, Std error = 0.04, p = 0.01) in the multivariable logistic regression model (Supplementary material S6).

### Trajectories of Cognitive Performance (Composite Cognitive Z-scores)

**Figure 3:**
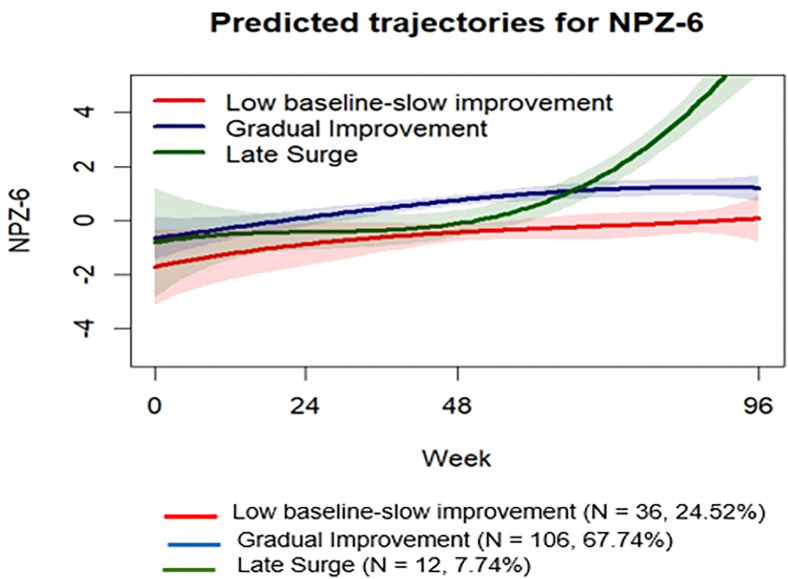
Predicted trajectory groups for cognitive z-score. “Low Baseline-Slow Improvement” group (24.52 %) is represented in red; “Gradual improvement” group (67.74%) is represented in blue; “Late Surge” group (7.74%) is represented in green. The shaded regions around the trajectories represent the 95% confidence intervals, illustrating the uncertainty or variability in the model’s predictions over time. Narrower shading indicates greater confidence in the predicted values. Clinically, the identification of a ‘Late Surge’ trajectory is of interest as it suggests that cognitive gains may manifest later in treatment for a subset of individuals, highlighting the potential for improvement even among those with initially limited progress.

Our analysis delineated three primary composite cognitive z-score trajectories that resonate with the patterns observed in our data. The best-fitting model was a three-group cubic model, reflecting the following patterns: “Low Baseline–Slow Improvement” (N = 36, 24.4%), “Gradual Improvement” (N = 106, 67.7%), and “Late Surge” (N = 12, 7.7%) (Figure 3). Supplementary Material S7 provides comprehensive details on the model’s goodness-of-fit and trajectory classification accuracy. While all three trajectories demonstrated improvement over 96 weeks post-ART, the “Low Baseline–Slow Improvement” group began with the lowest cognitive z-scores at baseline but showed steady gains, converging closely with the “Late Surge” group by approximately week 48.

Table 2 delineates the baseline characteristics distinguishing the cognitive trajectory groups. Notably, the “Low Baseline-Slow Improvement” group was significantly younger and had higher serum proBDNF levels compared to the “Gradual Improvement” group (p = 0.02). This group also displayed considerably lower composite cognitive z-scores relative to both the “Gradual Improvement” and “Late Surge” groups (p < 0.001). Multivariable logistic regression further identified baseline age (Log odds = −0.21, Std error = 0.07, p < 0.01) and baseline composite cognitive z-scores (Log odds = −1.89, Std error = 0.61, p < 0.001) as significant variables differentiating the “Low Baseline-Slow Improvement” group from the “Gradual Improvement” and “Late Surge” groups, underscoring the importance of these factors in predicting cognitive trajectory post-ART initiation (Supplementary material S8).

### Interrelationships Across the Trajectory Groups of Serum mBDNF and Cognitive performance

In examining the association between mBDNF trajectories and cognitive trajectories, distinct conditional probabilities emerged. Table 3a delineates the conditional probabilities of cognitive performance based on serum mBDNF trajectories. It shows individuals with a peak and decline mBDNF trajectory have a 68% probability of gradual cognitive improvement, with none exhibiting a late surge. Those with a stable ascent mBDNF trajectory show a 68% probability of gradual cognitive improvement, a lower probability of 22.52% for low baseline-slow improvement, and a 9.5% probability of a late surge.

Table 3b reflects the conditional probabilities of serum mBDNF trajectories given the cognitive trajectory groups. In the group with low baseline and slow improvement in composite cognitive z-scores, a 22.4% chance existed of following a peak and decline pattern in mBDNF levels, whereas a stable rise in mBDNF levels was highly probable at 77.6%. Within the gradual improvement cognitive trajectory, 16.9% are likely to have a peak and decline mBDNF trajectory, but a higher probability of 83.1% is observed for a stable ascent mBDNF trajectory. Notably, every individual within the late surge cognitive trajectory group is associated with a stable ascent mBDNF pattern, as indicated by the 100% probability.

Table 3c displays the joint probabilities for serum mBDNF and cognitive trajectories. The table highlights that individuals with a stable ascent mBDNF trajectory have the highest joint probability of 56.5% for being in the gradual improvement cognitive trajectory, a lower probability of 18.7% for the low baseline-slow improvement group, and 7.9% for the late surge group. For the peak and decline mBDNF trajectory, the joint probability is 5.4% for the low baseline-slow improvement group and 11.5% for the gradual improvement group, with a 0% probability for the late surge cognitive trajectory, indicating no occurrence of this mBDNF pattern in the late surge cognitive category.

### Interrelationships Across the Trajectory Groups of Serum proBDNF and Cognitive Performance

Table 4a provides the conditional probabilities of cognitive trajectories based on serum proBDNF levels. Individuals with a Gradual Decline in proBDNF have a 72.4% probability of falling into the Gradual improvement cognitive trajectory, while the probability of them experiencing a Late surge is 0%. For those with a Gradual Increase in proBDNF, there is a 67.7% probability for Gradual improvement and a notably lower 22.9% for Low Baseline-Slow improvement, with a small 9.4% for Late surge.

Table 4b shows the conditional probabilities of serum proBDNF trajectories given the cognitive trajectory group. In the Low Baseline-Slow improvement cognitive trajectory, 82.7% of individuals are characterized by a Gradual Increase in proBDNF, while only 17.3% show a Gradual Decline. The Gradual improvement cognitive group has a high probability of 84.4% for a Gradual Increase in proBDNF and a lower probability of 15.6% for a Gradual Decline. The Late surge trajectory is exclusively linked to a Gradual Increase in proBDNF, with a probability of 100%.

Table 4c presents the joint probabilities of serum proBDNF levels and cognitive trajectories. The highest joint probability is for a Gradual Increase in proBDNF with the Gradual improvement cognitive group at 57.7%, while the lowest is the absence of any individuals (0%) with a Gradual Decline in proBDNF in the Late surge cognitive trajectory. Additionally, there is a moderate joint probability of 19.5% for a Gradual Increase in proBDNF with the Low Baseline-Slow improvement cognitive group and a slight 8% with the Late surge group. The Gradual Decline proBDNF trajectory is least common, with a joint probability of 4.1% for the Low Baseline-Slow improvement group and 10.7% for the Gradual improvement group.

### Interrelationships Between Serum mBDNF and proBDNF Trajectories

Supplementary Material S9 provides detailed insights into the inter-relationship between serum mBDNF and proBDNF trajectories through conditional and joint probabilities. Table S7a presents the conditional probabilities of serum proBDNF trajectories based on mBDNF trajectories. Among individuals in the Stable Ascent mBDNF trajectory, 82.7% were associated with the Gradual Increase proBDNF trajectory, while 17.3% followed the Gradual Decline proBDNF trajectory. Conversely, for those in the Peak with Gradual Decline mBDNF trajectory, 86.2% belonged to the Gradual Decline proBDNF trajectory, and only 13.8% were aligned with the Gradual Increase proBDNF trajectory.

Table S7b reflects the reverse conditional probabilities of mBDNF trajectories given proBDNF trajectories. Individuals in the Gradual Decline proBDNF trajectory had a 27.6% probability of being in the Stable Ascent mBDNF trajectory and a 72.4% probability of following the Peak with Gradual Decline mBDNF trajectory. In contrast, among those in the Gradual Increase proBDNF trajectory, 77.1% were likely to belong to the Stable Ascent mBDNF trajectory, while 22.9% followed the Peak with Gradual Decline mBDNF trajectory.

Table S7c displays the joint probabilities of trajectory membership for serum mBDNF and proBDNF. The most frequent trajectory pairing was Stable Ascent mBDNF with a Gradual Increase in proBDNF, occurring in 70.3% of participants. This was followed by a Stable Ascent of mBDNF with a Gradual Decline in proBDNF (12.7%). The least common pairings were Peak with Gradual Decline mBDNF with either Gradual Increase proBDNF (2%) or Gradual Decline proBDNF (14.9%).

## DISCUSSION

This study is the first to examine the dynamic interplay between serum mBDNF and proBDNF and cognitive trajectories in people living with HIV after antiretroviral therapy initiation. Over 96 weeks, we identified two distinct serum mBDNF trajectories—stable ascent and peak with gradual decline—and two for proBDNF—gradual increase and gradual decline—each associated with three cognitive trajectories: low baseline with slow improvement, gradual improvement, and late surge. Notably, stable mBDNF ascent and gradual proBDNF increases were more commonly observed among participants with gradual cognitive improvement, while elevated proBDNF levels appeared in those with late cognitive surges. These findings suggest that mBDNF and proBDNF levels may be linked to neurocognitive improvement following ART initiation.

Our analysis reveals an association between serum mBDNF trajectories and cognitive trajectory, particularly noting that a stable ascent in mBDNF levels correlates with better cognitive trajectories. A stable ascent in mBDNF levels was predominantly observed in participants who demonstrated gradual cognitive improvement and in those experiencing late cognitive surges. This pattern may reflect the neurotrophic support provided by increasing mBDNF levels. Conversely, the trajectory of peaking followed by a gradual decline in mBDNF levels shows a lower likelihood of cognitive gains and is absent among those with late surges, suggesting its potential as an indicator of limited cognitive improvement. These observations are consistent with the proposed role of mBDNF in supporting neuroplasticity and counteracting neurodegeneration triggered by viral proteins[38–40].

The relationship between peripheral BDNF levels and central nervous system (CNS) function is complex. BDNF is primarily synthesized in the CNS but is also present peripherally and can cross the blood-brain barrier (BBB) in both directions[41]. Studies suggest that peripheral BDNF levels may reflect central BDNF activity, though this correlation can be influenced by various factors[42]. In the context of HIV, the virus can compromise BBB integrity, potentially altering BDNF transport dynamics[43]. Additionally, ART regimens have been associated with changes in BDNF signalling pathways[44]. While ART effectively reduces viral load and neuroinflammation, it may not fully restore BDNF signalling deficits, which may contribute to persistent neurocognitive challenges in people living with HIV[45]. These factors underscore the intricate interplay between peripheral BDNF dynamics, ART, and CNS neuroplasticity in PLWH.

This observation emphasizes the need for carefully designed and monitored cognitive enhancement strategies for people with HIV, particularly in settings with limited resources[46]. It points out the importance of methods that ensure a sustained increase in mBDNF levels for ongoing cognitive improvement, through various means such as exercise, nutritional supplements, or cognitive training[9, 47]. Evidence, including studies focused on HIV, shows that regular exercise boosts BDNF levels and response[48–50]. However, personalizing these interventions to match individual preferences, abilities, the nature and intensity of the activity, and considering time and resource availability, is essential for preserving these cognitive gains over time[51].

Unexpectedly, we found that “gradual increase” trajectories in serum proBDNF were linked to positive cognitive trajectories, diverging from our original hypothesis and the traditional understanding of proBDNF’s role in cognitive decline through mechanisms like long-term depression and neuronal atrophy[52, 53]. This finding contrasts with proBDNF’s known effects on spine retraction, dendritic pruning, and neuronal death[53–55]. However, aligning with our previous reports and supported by both preclinical and clinical studies, improved cognitive outcomes have been observed when increases in mBDNF are accompanied by rises in proBDNF levels[56–63]. This indicates that moderate increases in proBDNF levels could indicate ongoing neural remodelling and serve as a source for mBDNF production, potentially explaining its link to cognitive improvement[56]. Additionally, the beneficial impact of rising serum proBDNF levels might be most evident in the early phases of cognitive decline, suggesting a phase-specific role[58]. This emerging evidence suggests that the interaction between proBDNF and mBDNF in cognitive function may be more complex than the “yin-yang” hypothesis of opposing roles, leaning towards a nuanced understanding within the “continuum-sorting” hypothesis that emphasizes homeostatic balance[62, 63].

Across 96 weeks, specific covariates appeared to influence mBDNF, proBDNF, and cognitive trajectories. The stable ascent in mBDNF levels was more common among younger individuals, those with lower CD8 counts, and those with higher baseline mBDNF, suggesting a possible association between immunological status, neurotrophic support, and age. Younger individuals may possess greater neuroplastic capabilities, reflected in their mBDNF profiles[64, 65]. In addition, lower CD8 counts, indicative of reduced immune activation or inflammation, could provide a more conducive environment for mBDNF production and function[66]. Starting with higher mBDNF levels suggests an already established robust neurotrophic framework, essential for ongoing neuroplastic processes, potentially serving as a biomarker for cognitive reserve amidst HIV-associated cognitive challenges[67]. This relationship underscores the potential significance of considering biological and immunological factors in understanding mBDNF trajectories.

Our analysis revealed two main serum proBDNF trajectories: “Gradual Increase” and “Gradual Decline.” Study site emerged as a critical factor influencing these trajectories, suggesting that location-specific factors, possibly environmental enrichment or variations in clinical practices, may affect proBDNF dynamics. Logistic regression analysis further confirmed the study site’s pivotal role in determining proBDNF trajectories. These findings highlight the need to account for site-level confounders in future research on HIV-related cognitive outcomes[[68].

All cognitive trajectory groups in our study demonstrated improvement over time, consistent with prior evidence on the neurocognitive benefits of sustained ART in individuals with acute HIV infection [23]. Our analysis revealed that the “Low Baseline-Slow Improvement” group, characterized by a younger demographic and lower initial cognitive scores, showed a slower improvement trajectory. This was unexpected, as younger age is typically associated with greater neuroplasticity. One possible explanation is that younger individuals may face greater challenges with ART adherence, as reported in prior studies, which could blunt cognitive gains despite their biological advantage[69]. Given our cohort’s relatively young average age (39 years) and age-adjusted NPZ-6 scores, the data suggests that slightly older individuals within this young cohort may possess greater cognitive reserve, enabling them to achieve quicker cognitive gains post-ART. This reserve and an increased ability to develop compensatory mechanisms could explain the more rapid cognitive improvements observed post-ART in these slightly older individuals. This suggests a complex interplay between age, baseline cognitive status, and the trajectory of cognitive improvement in HIV[46, 70].

Our study’s methodology, employing a dual group-based modelling approach, represents a significant strength by probabilistically linking serum BDNF isoforms and cognitive performance trajectories, offering a nuanced view of longitudinal data. While the identified trajectories represent a spectrum of an underlying continuous phenomenon, caution is necessary for drawing causal inferences. The repeated measurement of serum mBDNF and proBDNF adds specificity to understanding their neurocognitive effects over time in people with HIV. However, the absence of tissue-level or neuroimaging data leaves the direct functional impact of BDNF on cognition uncertain. Future integration of neuroimaging could help clarify these mechanisms and link peripheral BDNF dynamics to brain-level activity.

These findings contribute to the understanding of BDNF dynamics in HIV-associated neurocognitive impairment. The stable ascent of mBDNF suggests its potential as a biomarker for identifying individuals at risk of cognitive decline or tracking improvement. Exploring how mBDNF and proBDNF interact with HIV-specific factors, such as chronic inflammation and immune activation, could guide personalized interventions to sustain cognitive improvement. Approaches like tailored exercise programs or nutritional strategies may enhance neuroplasticity and improve long-term outcomes.

### Limitations

The specific profile of our cohort— (young individuals living with HIV in a resource-limited setting, significantly immunosuppressed, part of a larger clinical trial and without significant comorbidities)—may restrict the generalizability of our findings. Moreover, since participants were recruited from two urban centers with relatively well-established clinical infrastructure, the applicability of these results to rural or less-resourced settings across sub-Saharan Africa may be limited. Additionally, a larger sample size may have unveiled more detailed group distinctions and supported more comprehensive models that could include a broader range of confounders. While our models accounted for several key demographic and clinical variables, there are additional covariates—such as inflammatory markers, ART adherence, psychosocial stressors, substance use history, ART regimen, depression, and nutritional status—that may influence BDNF expression and cognitive outcomes but were not included in this analysis. As such, residual confounding from unmeasured or excluded variables remains a possibility and may affect the interpretation of some observed relationships.

The neuropsychological battery employed in this study focused predominantly on motor and psychomotor speed, due to practical constraints related to testing time, staffing, and available infrastructure in the resource-limited setting. While these measures were feasible and clinically relevant, they do not capture higher-order cognitive domains such as memory, attention, and executive function, which are more frequently affected in HIV-associated neurocognitive disorders. This limited scope reduces the ability to fully interpret the cognitive significance of observed BDNF trajectories. Future studies should incorporate more comprehensive neurocognitive assessments and clinical criteria to better characterize the breadth of cognitive outcomes in this population.

Finally, relying on serum BDNF measurements rather than CNS-specific assessments limits the ability to directly infer CNS activity, as serum levels may not fully reflect central neurotrophic dynamics.

### Implications for Clinical Practice and Future Research

This study underscores the potential of mBDNF and proBDNF as biomarkers for monitoring cognitive trajectories in people with HIV. Clinically, the findings suggest prioritizing interventions that sustain mBDNF levels, such as tailored exercise programs, nutritional strategies, or cognitive training, which have shown promise in enhancing neuroplasticity. Personalized approaches are particularly crucial in resource-limited settings to maximize cognitive gains post-ART initiation.

Future research should validate these findings in larger, diverse cohorts and explore mechanisms linking peripheral BDNF dynamics with brain-level activity through neuroimaging and CNS-specific assessments. Additionally, the role of site-specific and biological factors, such as immune activation and inflammation, in shaping BDNF trajectories warrants further investigation to refine intervention strategies. Such efforts could lead to targeted, individualized care aimed at improving neurocognitive resilience and long-term outcomes in HIV populations.

## Data Availability

The authors confirm that all data are fully available upon request from sdac.data@sdac.harvard.edu with the written agreement of Advancing Clinical Therapeutics Globally for HIV/AIDS and Other Infections (ACTG). Study specimens were obtained from the ACTG Repository (www.specimenrepository.org).
